# Supplementary material for: Distinct and shared B cell responses of tuberculosis patients and their household contacts
Source: PLoS One. 2022 Oct 25;17(10):e0276610. doi: 10.1371/journal.pone.0276610 (PMC9595562; doi:10.1371/journal.pone.0276610)
Supplement: S1 Raw images — (PDF) [file pone.0276610.s001.pdf]

Supporting Information

Distinct and shared B cell responses of tuberculosis patients and their household contacts

Komal Singh, Rajesh Kumar, Fareha Umam, Prerna Kapoor, Sudhir Sinha, Amita Aggarwal

---

S1_raw_images. Original unprocessed images for Fig 3.

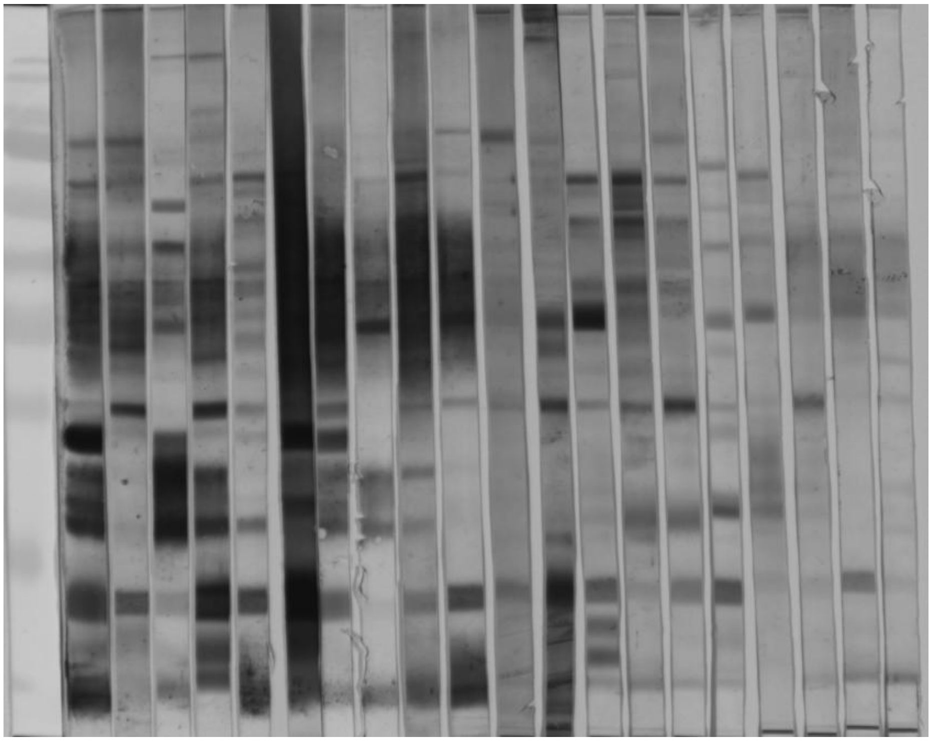

Original image for Figure 3 (left panel). 1st nitrocellulose paper (NCP) strip from left contains electroblotted, pre-stained molecular weight markers (PAGE Ruler Prestained Protein Ladder, Thermo Scientific, Cat No. 26616). 2nd to 21st NCP strips from left contain immunoblotting results with 20 TB sera (T1 to T20, Fig. 3). All strips are from the same gel and tiff image was acquired on ImageQuant LAS 500 (GE Healthcare).

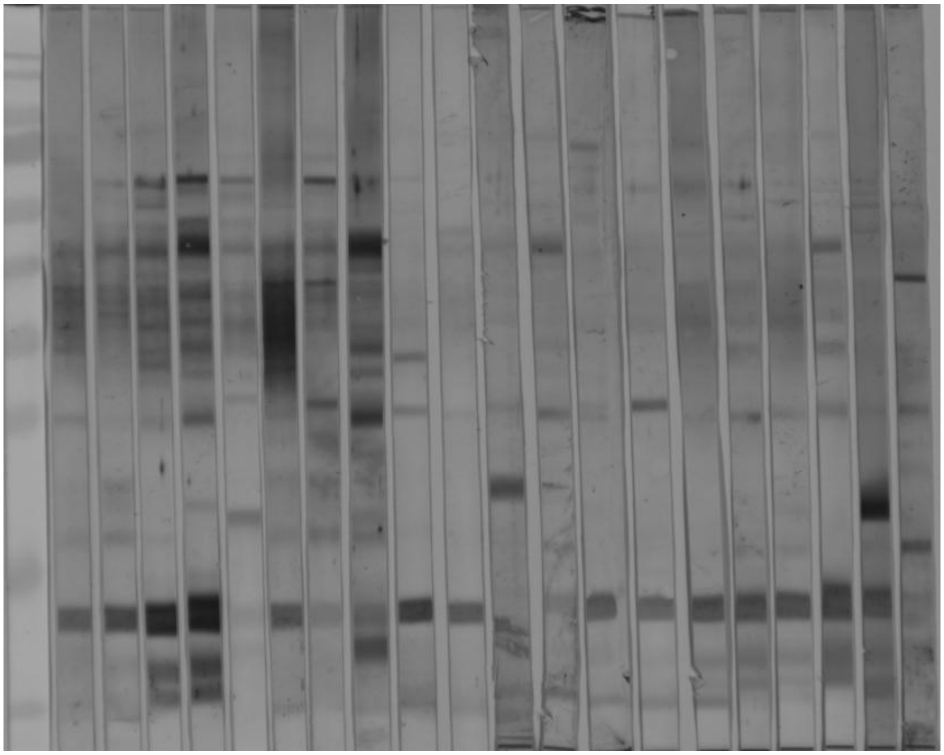

Original image for Figure 3 (right panel). 1st NCP strip from left contains electroblotted, pre-stained molecular weight markers (PAGE Ruler Prestained Protein Ladder, Thermo Scientific, Cat No. 26616). 2nd to 21st NCP strips from left contain immunoblotting results with 20 HHC sera (H1 to H20, Fig. 3). All strips are from the same gel and tiff image was acquired on ImageQuant LAS 500 (GE Healthcare).
