# Supplementary material for: Distinct and shared B cell responses of tuberculosis patients and their household contacts
Source: PLoS One. 2022 Oct 25;17(10):e0276610. doi: 10.1371/journal.pone.0276610 (PMC9595562; doi:10.1371/journal.pone.0276610)
Supplement: S1 Fig — (PDF) [file pone.0276610.s002.pdf]

Supporting Information

Distinct and shared B cell responses of tuberculosis patients and their household contacts

Komal Singh, Rajesh Kumar, Fareha Umam, Prerna Kapoor, Sudhir Sinha, Amita Aggarwal

---

S2 Figure. Relationship between tuberculin and B cell responses.

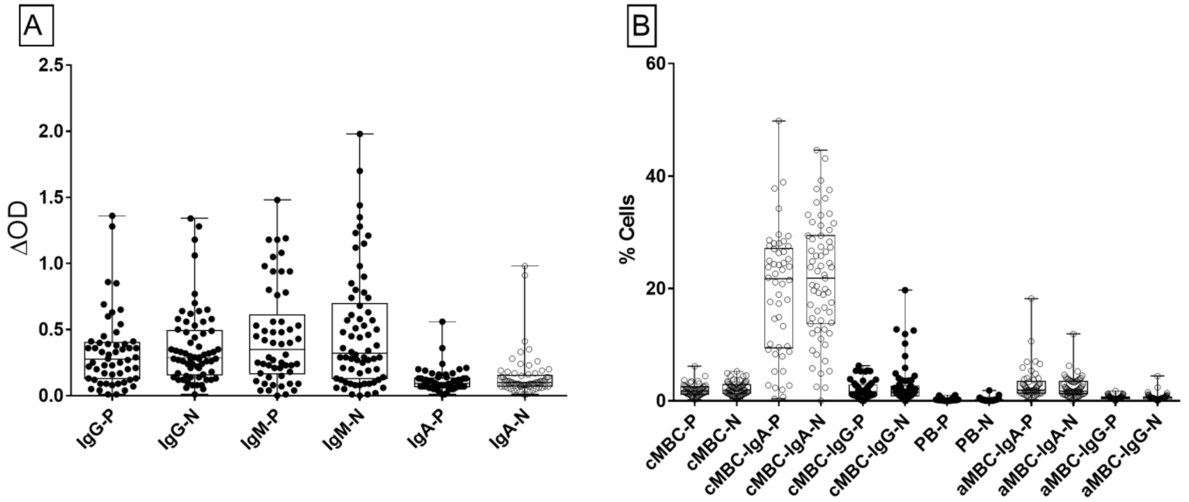

Panel A shows levels (ΔOD values) of IgG, IgM and IgA antibodies to MtM and panel B shows frequencies of various B cell subsets in HHCs who were either positive (P) or negative (N) for TST. For each parameter, the difference between P and N subgroups was not significant ($P > 0.05$).
