# Supplementary material for: Distinct and shared B cell responses of tuberculosis patients and their household contacts
Source: PLoS One. 2022 Oct 25;17(10):e0276610. doi: 10.1371/journal.pone.0276610 (PMC9595562; doi:10.1371/journal.pone.0276610)
Supplement: S1 Table — (PDF) [file pone.0276610.s003.pdf]

Supporting Information

Distinct and shared B cell responses of tuberculosis patients and their household contacts

Komal Singh, Rajesh Kumar, Fareha Umam, Prerna Kapoor, Sudhir Sinha, Amita Aggarwal

S3 Table. Raw data for Fig 1, 2, 5 and 6.

Raw data for Figure 1A and Figure 2

IgG [HHC]	IgG [TB]	IgM [HHC]	IgM [TB]	IgA [HHC]	IgA [TB]
0.04	0.29	0.39	0.13	0.05	0.14
0.24	0.12	0.10	0.24	0.07	0.01
0.26	0.26	0.09	0.45	0.03	0.13
0.28	1.15	0.25	0.99	0.10	1.56
0.28	0.79	0.63	0.23	0.11	0.28
0.32	1.20	0.06	0.90	0.03	1.60
0.34	1.06	0.09	0.60	0.09	0.46
0.01	0.36	0.27	0.31	0.08	0.21
0.29	1.01	0.27	0.04	0.06	0.22
0.26	0.83	0.38	0.16	0.14	0.13
0.35	0.52	0.80	0.14	0.11	0.11
0.69	0.32	0.94	0.20	0.11	0.03
0.36	0.99	0.21	0.28	0.07	0.07
0.31	1.61	0.27	0.57	0.07	1.84
0.29	0.22	0.18	0.57	0.13	0.14
0.21	1.15	0.48	0.68	0.11	0.33
0.12	1.15	0.14	0.54	0.14	0.22
0.12	1.33	0.29	0.96	0.08	0.36
0.06	0.98	0.20	1.25	0.07	0.06
0.12	0.71	0.20	0.72	0.08	0.18
0.10	1.34	0.09	1.27	0.21	0.30
0.09	1.15	0.01	0.48	0.02	0.61
0.85	1.33	0.25	0.92	0.12	1.67
0.62	0.78	0.02	0.36	0.07	0.18
0.20	1.24	0.15	1.10	0.16	0.25
1.28	0.50	0.36	0.58	0.98	0.23
0.36	0.94	0.04	1.11	0.56	0.57
0.01	0.71	0.05	0.27	0.04	0.16
0.40	1.35	0.76	0.26	0.19	0.22
0.33	1.38	0.08	0.95	0.01	1.78
0.13	1.10	0.53	0.75	0.08	0.34
0.28	1.49	0.00	0.24	0.05	0.80

0.63	1.39	0.50	0.49	0.11	2.39
0.05	0.29	0.01	0.96	0.06	0.84
0.14	0.46	0.29	0.25	0.05	0.17
0.39	0.69	0.25	0.64	0.12	0.12
0.23	0.87	0.11	0.23	0.05	0.20
0.01	0.20	0.14	0.49	0.01	0.16
0.39	2.47	0.31	2.07	0.07	1.59
1.28	1.59	0.98	0.68	0.36	0.08
0.26	0.71	0.44	0.86	0.24	0.13
0.09	1.22	0.40	0.28	0.13	0.04
0.37	0.92	0.28	0.14	0.33	0.02
0.17	0.39	0.56	0.59	0.13	0.09
0.41	1.56	0.48	0.21	0.14	0.06
0.86	2.17	1.48	1.45	0.17	0.08
0.36	1.71	0.53	0.32	0.20	1.02
0.22	0.28	0.58	1.19	0.10	0.14
0.30	2.28	0.74	0.15	0.10	0.38
0.17	1.23	0.23	0.36	0.07	0.16
1.36	0.94	0.24	0.13	0.16	0.14
0.35	1.39	0.94	0.17	0.12	0.04
0.16		0.12		0.01	
0.24	1.04	0.19	0.01	0.07	2.85
0.48	2.73	0.43	0.32	0.08	3.00
0.27		0.85		0.28	
0.56		0.98		0.10	
0.13		0.80		0.01	
0.29		1.28		0.41	
0.54		0.24		0.07	
0.31		1.18		0.13	
0.33		0.23		0.14	
0.05		1.18		0.20	
0.15		0.68		0.08	
0.41		0.56		0.09	
0.14		1.70		0.11	
0.12		1.19		0.11	
0.28		0.50		0.09	
0.32		0.90		0.09	
0.07		0.94		0.09	
0.18		0.13		0.08	
0.70		0.61		0.35	
0.49		0.74		0.18	
0.58		0.53		0.12	
0.44		0.57		0.09	
0.47		1.23		0.19	
0.65		0.45		0.04	

1.06	0.01	0.04
0.25	0.21	0.05
0.58	0.10	0.09
0.24	0.29	0.11
0.09	0.78	0.08
0.08	0.42	0.07
0.36	0.09	0.06
0.04	0.23	0.07
0.33	0.04	0.06
0.16	0.40	0.05
0.08	0.33	0.04
0.08	1.35	0.14
0.21	0.78	0.16
0.12	1.15	0.10
0.25	1.05	0.09
0.35	1.12	0.09
0.13	1.44	0.07
0.60	0.04	0.06
0.28	0.49	0.24
0.38	0.29	0.10
0.22	0.08	0.05
0.20	0.00	0.02
0.21	1.08	0.17
0.27	0.07	0.07
0.12	0.09	0.11
0.65	0.12	0.09
0.50	0.29	0.23
1.34	0.45	0.14
1.18	0.17	0.26
0.14	0.06	0.08
0.10	0.21	0.11
0.08	0.57	0.17
0.23	0.48	0.12
0.45	0.17	0.07
0.77	1.98	0.15
0.64	0.34	0.91
0.53	0.28	0.19
0.40	0.31	0.17
0.33	0.46	0.09
0.48	0.08	0.08
0.17	0.09	0.06
0.64	0.51	0.12
0.56	1.21	0.20

Raw data for Figure 1B

HHC	TB
9.75	0.45
0.42	2.00
0.35	1.73
0.89	0.86
2.25	0.29
0.19	0.75
0.26	0.57
27.00	0.86
0.93	0.04
1.46	0.19
2.29	0.27
1.36	0.63
0.58	0.28
0.87	0.35
0.62	2.59
2.29	0.59
1.17	0.47
2.42	0.72
3.33	1.28
1.67	1.01
0.90	0.95
0.11	0.42
0.29	0.69
0.03	0.46
0.75	0.89
0.28	1.16
0.11	1.18
5.00	0.38
1.90	0.19
0.24	0.69
4.08	0.68
0.04	0.16
0.79	0.35
0.20	3.31
2.07	0.54
0.64	0.93
0.48	0.26
14.00	2.45
0.79	0.84
0.77	0.43

1.69	1.21
4.44	0.23
0.76	0.15
3.29	1.51
1.17	0.13
1.72	0.67
1.47	0.19
2.64	4.25
2.47	0.07
1.35	0.29
0.18	0.14
2.69	0.12
0.75	
0.79	0.01
0.90	0.12
3.15	
1.75	
6.15	
4.41	
0.44	
3.81	
0.70	
23.60	
4.53	
1.37	
12.14	
9.92	
1.79	
2.81	
13.43	
0.72	
0.87	
1.51	
0.91	
1.30	
2.62	
0.69	
0.01	
0.84	
0.17	
1.21	
8.67	
5.25	

0.25
5.75
0.12
2.50
4.13
16.88
3.71
9.58
4.20
3.20
11.08
0.07
1.75
0.76
0.36
0.05
5.14
0.26
0.75
0.18
0.58
0.34
0.14
0.43
2.10
7.13
2.09
0.38
2.57
0.53
0.53
0.78
1.39
0.17
0.53
0.80
2.16

Raw data for Figure 5A

cMBC [HHC]	cMBC [TB]	IgA+ cMBC [HHC]	IgA+ cMBC [TB]	IgG+ cMBC [HHC]	IgG+ cMBC [TB]	PB [HHC]	PB [TB]
2.25	0.41	21.10	58.70	5.31	6.59	0.33	0.21
0.89	0.51	29.40	22.40	2.03	10.40	0.10	0.29
1.99	1.25	26.70	66.10	4.04	4.97	0.04	0.04
0.78	1.97	37.50	26.80	19.70	1.89	0.07	0.09
1.34	0.77	43.10	41.40	7.97	7.21	0.23	0.41
1.00	3.39	19.20	14.40	3.61	2.97	0.08	0.17
1.55	0.95	31.80	24.10	0.85	2.76	0.10	0.01
1.29	1.82	24.50	40.00	5.28	2.00	0.15	0.31
2.56		23.80		0.50		0.29	
0.75		35.30		2.63		0.15	
0.58	0.28	14.70	40.70	3.68	4.88	0.17	1.52
0.57	0.30	9.68	39.70	0.01	4.11	0.15	0.01
0.74	0.96	23.80	33.30	2.38	4.44	0.01	0.01
	2.25		23.70		13.80		0.30
1.43	1.55	18.00	23.70	1.47	2.40	0.20	0.39
2.87	0.38	37.70	42.10	1.19	3.01	0.21	0.01
2.97	0.75	28.80	69.30	1.48	0.99	0.22	0.01
1.02	0.92	39.20	24.60	2.43	1.69	0.03	2.77
1.32	1.09	33.10	28.10	1.31	0.78	0.04	0.41
0.72	1.33	33.30	38.40	0.39	2.80	0.20	0.32
1.65	0.33	49.80	66.70	3.77	0.01	0.13	0.23
0.98	1.65	27.00	48.40	5.40	4.69	0.92	0.32
1.11	0.52	23.30	35.80	2.56	3.67	0.19	0.18
2.89	2.60	33.00	38.30	3.56	1.09	0.53	0.15
2.34	2.06	38.90	31.90	0.84	2.35	0.16	0.49
2.19	0.86	44.60	25.80	1.50	1.03	0.49	0.63
0.19	0.93	8.33	33.60	0.01	0.70	0.65	0.01
0.66		24.80		11.90		0.44	
2.77	0.25	24.90	6.67	1.94	20.00	0.18	0.88
2.09	0.62	21.80	5.56	4.46	13.00	0.07	0.03
3.65	0.88	27.60	15.90	2.13	3.18	0.35	0.01
5.25	0.24	25.80	14.90	12.50	2.99	0.10	0.06
1.95	0.39	25.20	16.30	1.25	1.16	0.14	0.11
2.28	1.78	19.60	18.10	0.80	5.48	0.06	0.02
1.41	0.80	27.30	8.97	1.15	10.90	0.24	0.96
1.12	0.98	26.30	26.50	2.02	2.24	0.29	0.28
0.50	0.75	27.50	16.50	6.25	4.40	0.13	0.06
1.19	2.43	34.20	31.20	1.75	5.10	0.12	2.38
1.50	1.95	17.60	6.98	1.84	3.36	0.01	3.10
	2.12		18.10		3.61		0.62
2.20	0.99	28.30	11.40	2.09	0.40	0.20	0.28

1.91	0.37	28.40	3.61	3.86	4.82	0.32	0.08
4.44	1.03	36.00	5.71	0.38	10.50	0.36	1.08
2.13		14.60		2.81		0.29	
0.86		26.50		1.02		0.11	
2.51		24.20		5.23		0.17	
1.87	1.12	18.90	2.04	5.36	2.04	0.01	0.15
1.54		7.08		1.77		0.23	
2.85	1.65	5.25	17.40	0.71	5.56	0.03	0.72
1.15	1.17	13.30	4.66	0.51	2.07	0.01	0.45
3.23	0.80	9.11	2.40	0.71	1.03	0.21	0.15
1.13	0.61	8.20	4.84	1.64	1.61	0.01	0.12
1.31	1.72	24.50	7.39	2.53	2.82	0.03	0.28
4.39	0.22	2.30	7.14	0.92	14.30	0.08	0.07
3.29		24.00		0.88		0.09	
1.30		20.40		1.53		0.14	
2.21		7.90		2.27		0.12	
3.75		5.78		3.65		0.46	
1.30		37.80		1.46		0.09	
2.07		28.00		1.62		0.10	
1.53		17.30		1.16		0.08	
2.61		22.60		1.14		0.12	
4.50		12.60		0.92		0.55	
1.82		2.73		1.02		0.10	
1.88		4.84		2.90		0.27	
1.11		23.80		1.55		0.09	
0.51		20.60		5.88		0.01	
0.47		5.21		1.63		1.00	
1.84		12.00		2.29		0.08	
2.60		16.00		1.60		0.05	
3.47		14.30		0.57		0.36	
2.62		23.10		0.94		0.11	
3.83		17.10		0.66		0.08	
1.88		16.60		0.92		0.06	
0.93		8.76		2.99		0.96	
1.69		5.28		1.51		0.24	
1.61		10.10		0.31		0.01	
4.91		12.00		0.77		0.11	
2.23		18.90		2.04		0.20	
2.00		9.51		2.76		0.51	
1.82		21.50		0.79		0.22	
2.54		29.60		3.15		0.16	
2.02		23.90		1.54		0.04	
0.48		28.00		2.67		0.80	
2.49		20.80		1.08		0.60	
0.36		29.40		2.10		0.19	

4.40	0.01	0.60	0.35
1.65	13.90	0.32	0.21
1.82	31.60	0.01	1.84
1.49	2.75	3.57	0.40
1.58	31.20	0.01	0.55
2.63	2.34	1.17	0.22
3.49	0.36	0.24	0.09
3.45	0.69	0.23	0.20
1.37	1.96	0.98	0.13
1.23	29.30	0.95	0.01
1.58	21.90	2.11	0.06
6.15	24.30	1.62	0.01
3.65	22.40	0.68	0.08
1.87	15.80	1.84	0.06
1.68	19.90	1.74	0.08
1.65	2.51	1.92	0.09
2.32	17.50	1.41	1.05
2.74	27.40	1.58	0.13
0.62	14.90	3.09	0.17
3.07	28.60	2.74	0.36
3.11	19.30	0.70	0.03
1.17	9.98	4.87	0.06
3.26	13.70	2.14	0.11
2.39	8.97	1.03	0.02
0.85	11.00	2.05	0.01
1.38	25.80	1.51	0.05
1.10	26.50	10.20	0.16
1.77	30.40	0.74	0.16
3.43	12.70	12.70	0.35

Raw data for Figure 5B

IgA+ aMBC [HHC]	IgA+ aMBC [TB]	IgG+ aMBC [HHC]	IgG+ aMBC [TB]
1.78	3.57	0.68	0.96
2.99	2.95	2.40	0.84
3.85	4.18	0.55	1.27
0.78	2.83	0.26	0.39
3.29	1.16	0.74	0.64
3.44	3.68	0.77	1.03
4.62	1.74	0.40	0.51
4.04	1.53	0.48	1.78
4.08		0.57	
3.93		0.41	
0.78	5.83	0.52	0.38
0.95	3.09	1.26	0.18
1.62	4.34	0.51	0.84
	3.43		0.27
5.35	3.63	0.63	0.76
6.23	0.15	0.24	1.55
1.76	9.45	0.37	0.30
1.91	7.71	0.59	1.09
1.30	3.19	0.52	0.39
1.57	4.13	0.74	0.46
1.19	1.86	0.33	1.03
1.94	6.18	0.41	1.00
3.82	1.03	0.79	0.60
5.25	11.90	1.00	0.66
2.67	14.00	0.64	0.73
3.89	3.15	0.19	0.81
6.93	1.53	0.99	0.74
2.04		0.46	
1.81	1.28	0.35	0.12
3.30	0.27	0.74	0.41
2.44	3.91	0.98	0.78
3.47	1.29	4.40	0.32
0.64	1.20	0.59	0.35
4.48	1.64	0.98	0.23
4.18	1.26	0.29	0.45
2.75	1.42	0.51	0.17
5.91	0.93	0.33	0.62
3.07	7.22	0.52	1.26
2.97	1.05	0.90	0.32
	1.63		0.32
11.90	0.84	0.63	0.44

3.68	0.67	0.70	0.46
3.49	1.84	0.45	0.20
1.81		0.69	
2.38		1.27	
1.23		1.11	
6.58	0.21	0.18	1.45
3.74		0.47	
1.94	1.11	0.52	0.26
1.25	1.22	0.45	0.97
4.79	0.97	1.78	0.48
1.93	0.81	0.77	0.54
1.82	3.82	0.81	0.62
3.37	0.29	0.35	0.66
2.62		0.44	
2.95		0.67	
1.86		0.71	
1.55		0.52	
2.30		0.40	
1.80		0.79	
1.20		1.20	
2.92		0.33	
2.18		0.97	
1.66		0.22	
1.00		0.51	
2.05		0.25	
0.52		0.01	
1.37		0.42	
0.59		0.59	
1.06		0.54	
0.68		0.16	
1.24		0.40	
1.24		0.56	
1.38		0.18	
1.36		0.64	
1.70		0.28	
2.89		0.78	
1.18		0.14	
1.55		0.15	
0.98		0.55	
1.63		0.16	
0.90		0.14	
2.45		0.33	
18.20		0.00	
7.01		0.64	
1.67		0.40	

0.01	0.31
1.75	0.10
1.52	1.52
1.79	0.45
3.70	0.60
0.66	0.24
0.68	0.45
0.23	0.10
0.54	0.54
1.96	0.41
1.84	0.44
10.60	0.50
2.27	0.65
0.79	0.51
1.14	0.52
1.50	1.36
2.35	0.34
2.48	0.30
1.33	0.37
4.00	0.34
1.65	0.63
0.77	0.37
1.51	0.36
1.04	0.32
2.41	0.56
0.71	1.04
1.58	1.11
3.58	0.49
1.62	0.42

Raw data for Figure 6

CD27+IgA+ [HHC]	CD27-IgA+ [HHC]	CD27+IgG+ [HHC]	CD27-IgG+ [HHC]	CD27+IgA+ [TB]	CD27-IgA+ [TB]	CD27+IgG+ [TB}	CD27-IgG+ [TB]
21.10	1.78	5.31	0.68	58.70	3.57	6.59	0.96
29.40	2.99	2.03	2.40	22.40	2.95	10.40	0.84
26.70	3.85	4.04	0.55	66.10	4.18	4.97	1.27
37.50	0.78	19.70	0.26	26.80	2.83	1.89	0.39
43.10	3.29	7.97	0.74	41.40	1.16	7.21	0.64
19.20	3.44	3.61	0.77	14.40	3.68	2.97	1.03
31.80	4.62	0.85	0.40	24.10	1.74	2.76	0.51
24.50	4.04	5.28	0.48	40.00	1.53	2.00	1.78
23.80	4.08	0.50	0.57				
35.30	3.93	2.63	0.41				
14.70	0.78	3.68	0.52	40.70	5.83	4.88	0.38
9.68	0.95	0.01	1.26	39.70	3.09	4.11	0.18
23.80	1.62	2.38	0.51	33.30	4.34	4.44	0.84
				23.70	3.43	13.80	0.27
18.00	5.35	1.47	0.63	23.70	3.63	2.40	0.76
37.70	6.23	1.19	0.24	42.10	0.15	3.01	1.55
28.80	1.76	1.48	0.37	69.30	9.45	0.99	0.30
39.20	1.91	2.43	0.59	24.60	7.71	1.69	1.09
33.10	1.30	1.31	0.52	28.10	3.19	0.78	0.39
33.30	1.57	0.39	0.74	38.40	4.13	2.80	0.46
49.80	1.19	3.77	0.33	66.70	1.86	0.01	1.03
27.00	1.94	5.40	0.41	48.40	6.18	4.69	1.00
23.30	3.82	2.56	0.79	35.80	1.03	3.67	0.60
33.00	5.25	3.56	1.00	38.30	11.90	1.09	0.66
38.90	2.67	0.84	0.64	31.90	14.00	2.35	0.73
44.60	3.89	1.50	0.19	25.80	3.15	1.03	0.81
8.33	6.93	0.01	0.99	33.60	1.53	0.70	0.74
24.80	2.04	11.90	0.46				
24.90	1.81	1.94	0.35	6.67	1.28	20.00	0.12
21.80	3.30	4.46	0.74	5.56	0.27	13.00	0.41
27.60	2.44	2.13	0.98	15.90	3.91	3.18	0.78
25.80	3.47	12.50	4.40	14.90	1.29	2.99	0.32
25.20	0.64	1.25	0.59	16.30	1.20	1.16	0.35
19.60	4.48	0.80	0.98	18.10	1.64	5.48	0.23
27.30	4.18	1.15	0.29	8.97	1.26	10.90	0.45
26.30	2.75	2.02	0.51	26.50	1.42	2.24	0.17
27.50	5.91	6.25	0.33	16.50	0.93	4.40	0.62
34.20	3.07	1.75	0.52	31.20	7.22	5.10	1.26
17.60	2.97	1.84	0.90	6.98	1.05	3.36	0.32
				18.10	1.63	3.61	0.32
28.30	11.90	2.09	0.63	11.40	0.84	0.40	0.44

28.40	3.68	3.86	0.70	3.61	0.67	4.82	0.46
36.00	3.49	0.38	0.45	5.71	1.84	10.50	0.20
14.60	1.81	2.81	0.69				
26.50	2.38	1.02	1.27				
24.20	1.23	5.23	1.11				
18.90	6.58	5.36	0.18	2.04	0.21	2.04	1.45
7.08	3.74	1.77	0.47				
5.25	1.94	0.71	0.52	17.40	1.11	5.56	0.26
13.30	1.25	0.51	0.45	4.66	1.22	2.07	0.97
9.11	4.79	0.71	1.78	2.40	0.97	1.03	0.48
8.20	1.93	1.64	0.77				
24.50	1.82	2.53	0.81	7.39	3.82	2.82	0.62
2.30	3.37	0.92	0.35	7.14	0.29	14.30	0.66
24.00	2.62	0.88	0.44				
20.40	2.95	1.53	0.67				
7.90	1.86	2.27	0.71				
5.78	1.55	3.65	0.52				
37.80	2.30	1.46	0.40				
28.00	1.80	1.62	0.79				
17.30	1.20	1.16	1.20				
22.60	2.92	1.14	0.33				
12.60	2.18	0.92	0.97				
2.73	1.66	1.02	0.22				
4.84	1.00	2.90	0.51				
23.80	2.05	1.55	0.25				
20.60	0.52	5.88	0.01				
5.21	1.37	1.63	0.42				
12.00	0.59	2.29	0.59				
16.00	1.06	1.60	0.54				
14.30	0.68	0.57	0.16				
23.10	1.24	0.94	0.40				
17.10	1.24	0.66	0.56				
16.60	1.38	0.92	0.18				
8.76	1.36	2.99	0.64				
5.28	1.70	1.51	0.28				
10.10	2.89	0.31	0.78				
12.00	1.18	0.77	0.14				
18.90	1.55	2.04	0.15				
9.51	0.98	2.76	0.55				
21.50	1.63	0.79	0.16				
29.60	0.90	3.15	0.14				
23.90	2.45	1.54	0.33				
28.00	18.20	2.67	0.00				
20.80	7.01	1.08	0.64				
29.40	1.67	2.10	0.40				

0.01	0.01	0.60	0.31
13.90	1.75	0.32	0.10
31.60	1.52	0.01	1.52
2.75	1.79	3.57	0.45
31.20	3.70	0.01	0.60
2.34	0.66	1.17	0.24
0.36	0.68	0.24	0.45
0.69	0.23	0.23	0.10
1.96	0.54	0.98	0.54
29.30	1.96	0.95	0.41
21.90	1.84	2.11	0.44
24.30	10.60	1.62	0.50
22.40	2.27	0.68	0.65
15.80	0.79	1.84	0.51
19.90	1.14	1.74	0.52
2.51	1.50	1.92	1.36
17.50	2.35	1.41	0.34
27.40	2.48	1.58	0.30
14.90	1.33	3.09	0.37
28.60	4.00	2.74	0.34
19.30	1.65	0.70	0.63
9.98	0.77	4.87	0.37
13.70	1.51	2.14	0.36
8.97	1.04	1.03	0.32
11.00	2.41	2.05	0.56
25.80	0.71	1.51	1.04
26.50	1.58	10.20	1.11
30.40	3.58	0.74	0.49
12.70	1.62	12.70	0.42
